# Influence of *Smallanthus sonchifolius* (Yacon) on the Activity of Antidepressant Drugs in Mice

**DOI:** 10.3390/life11111117

**Published:** 2021-10-21

**Authors:** Sylwia Wośko, Anna Serefko, Aleksandra Szopa, Sylwia Kardaś, Jarosław Widelski, Tomasz Mroczek, Ewelina Rostkowska, Jolanta Szymańska, Ewa Poleszak

**Affiliations:** 1 Laboratory of Preclinical Testing, Chair and Department of Applied and Social Pharmacy, Medical University of Lublin, Chodźki 1, 20-093 Lublin, Poland; sylwia.wosko@umlub.pl (S.W.); ewa.poleszak@umlub.pl (E.P.); 2 Student’s Scientific Circle at Laboratory of Preclinical Testing, Medical University of Lublin, Chodźki 1, 20-093 Lublin, Poland; 54187@student.umlub.pl; 3 Department of Pharmacognosy, Medical University of Lublin, Chodźki 1, 20-093 Lublin, Poland; jaroslaw.widelski@umlub.pl; 4 Independent Laboratory of Chemistry of Natural Products, Medical University of Lublin, Chodźki 1, 20-093 Lublin, Poland; tomasz.mroczek@umlub.pl; 5 Beauty Salon “Clarie Beauty”, Pułaskiego 26, 20-461 Lublin, Poland; farm.stos@umlub.pl; 6 Department of Integrated Paediatric Dentistry, Medical University of Lublin, Chodźki 6, 20-093 Lublin, Poland; jolanta.szymanska@umlub.pl

**Keywords:** Yacon (*Smallantchus sonchifolius*), imipramine, fluoxetine, reboxetine, forced swim test, mice

## Abstract

Depression is one of the most common mental disorders in the world that negatively affects the daily functioning of patients. Numerous studies are currently being conducted to examine the antidepressant potential of innovative synthetic compounds and herbal substances. Yacon, *Smallantchus sonchifolius*, belongs to plants with numerous health-beneficial properties. Yacon-based products are regarded as a functional food. In our study, we attempted to check whether administration of Yacon tuber extract would have an antidepressant effect in the forced swim test (FST) in mice and whether its intake could influence the activity of conventional antidepressant drugs with different mechanisms of action, i.e., imipramine hydrochloride, fluoxetine hydrochloride, and reboxetine mesylate. The spontaneous locomotor activity of the tested mice was also investigated to eliminate any false-positive results. We demonstrated that an intragastric administration of the Yacon tuber extract at a dose of 100 mg/kg induced the antidepressant-like behavior in the FST in mice and that a combined administration of the sub-effective doses of the Yacon extract (50 mg/kg) with imipramine hydrochloride (7.5 mg/kg), fluoxetine hydrochloride (20 mg/kg), or reboxetine mesylate (5 mg/kg) significantly reduced the immobility time of animals in this behavioral test. The obtained results were not affected by the increased locomotor activity of the tested subjects. In conclusion, our findings suggest that Yacon tuber extract is promising as an alternative mood-improving product since it possesses an antidepressant potential and it can acts synergistically with conventional antidepressant drugs.

## 1. Introduction

Over the years, there has been a tendency of increased incidence of depression. In addition to biological factors, currently prevailing lifestyle, insecurity, social alienation, and associated stress are indicated as the main causes of this phenomenon. Depression is a common disease that, according to the WHO report, affects approximately 355 million people worldwide [1]. Despite advances in the diagnosis and treatment of depressive disorders, the number of suicides in the world continues to increase, which is a serious problem, not only clinically but also socially [2]. Due to its complex pathomechanism, depression is a disease difficult to treat. Drug choices depend on a clinical picture of depression, presence of comorbidities, the onset of antidepressant action, and safety profile of a given agent (side effects, toxicity, interactions). Pharmacotherapy of depressive disorders began over 50 years ago when tricyclic antidepressants (TCAs) and monoamine oxidase inhibitors (MAOIs) were discovered [3]. Later on, a newer generation of antidepressants was introduced, which included: selective serotonin reuptake inhibitors (SSRIs), selective serotonin and norepinephrine reuptake inhibitors (SNRIs), selective norepinephrine reuptake inhibitors (NRIs), selective norepinephrine and dopamine reuptake inhibitors (NDRIs), and atypical antidepressants. However, pharmacotherapy for mood disorders still does not produce satisfactory results. It has been estimated that 30–40% of depressed patients do not experience any satisfactory effects of the prescribed therapy, and over 50–70% of patients do not achieve full remission [4]. Moreover, delayed onset of antidepressant action (ca. 6–8 weeks) and occurrence of treatment-associated side effects upset patients who, as a result, stop taking medications [5,6]. Thus, there is a great need to develop new treatment strategies with a faster onset of action that will produce a complete remission of symptoms, will not induce side effects, and will not negatively interact with other active substances.

A plant that is currently attracting much attention because of its health-promoting properties, including the antidepressant one, is Yacon–*Smallantchus sonchifolius*, considered as a functional food [7]. It belongs to the *Asteraceae* family, and it can be found in the Andean region of South America. Yacon roots contain minerals (zinc, phosphorus, iron, copper, calcium), vitamins (B1, B2, B3, C), tryptofan, proteins, lipids, polyphenols (such as protocatechuic acid, chlorogenic acid, caffeic acid, and ferulic acid), and high amounts of indigestible oligosaccharides (such as fructooligosaccharides and inulin) [8,9,10,11,12]. In folk medicine, Yacon is regarded as a remedy for diabetes, rachitis, liver and kidney diseases, obesity, digestion problems. It is also used as a skin rejuvenator [7]. Some of these therapeutic properties have been confirmed in preclinical and clinical studies. Researchers have documented an antimicrobial [13,14], hypoglycemic [15], antiobesity [16], anticancer [17,18], and cholesterol-lowering activity of Yacon preparations in animals models and in in vitro experiments. It has also been found out that the main substances detected in Yacon roots and leaves exert antioxidant (polyphenols, tryptophan), anti-inflammatory (oligosaccharides, polyphenols) [19,20,21,22], and prebiotic effects (fructooligosaccharides, inulin) [20,21] that contribute to the above-mentioned health-beneficial properties of Yacon. In clinical trials, Yacon-based products increased defecation frequency, reduced weight, and elevated fasting serum insulin [23], enhanced immunity [24], had a favorable effect on serum glucose [25], improved body composition and intestinal function [26], increased satiety [27], and reduced constipation symptoms [28].

Several years ago, an and colleagues [29] reported that inulin-type oligosaccharides extracted from Yacon root possess an antidepressant-like potential. Furthermore, the authors mentioned that a similar type of oligosaccharides, extracted from *Morinda officinalis*, also had exerted the antidepressant-like activity in animal models [30,31]. Moreover, it has been suggested that the inulin-type hexasaccharide obtained from *Morinda officinalis* could produce neuroprotective effects [32]. In view of the above, we decided to check whether administration of the whole Yacon tuber extract would also have an antidepressant-like effect and whether its intake could influence the activity of antidepressant drugs. The whole Yacon tuber extract contains not only fructooligosaccharides but also many other biologically active substances (mentioned above), which may interact with each other. Therefore the biological effect of the whole Yacon tuber extract represents a combined activity of all its active ingredients. Our study was carried out in albino Swiss mice with the use of three standard antidepressants with distinct mechanisms of action: imipramine belonging to TCAs, fluoxetine belonging to SSRIs, and reboxetine belonging to NRIs. At first, we wanted to investigate whether the main substances in Yacon tuber extract (particularly fructooligosaccharides but also other components) do not have a negative impact on the absorption of antidepressants. Fructooligosaccharides are not metabolized by humans, they are partially fermented by the colonic bacteria, and they may influence the absorption of other compounds in the alimentary system [33]. After that, we wanted to check whether it is possible to obtain a synergistic antidepressant effect by a concomitant intake of Yacon tuber extract and antidepressant agents. The possibility of using the whole Yacon tuber extract in the treatment of depression as an addition to the conventional treatment would be very attractive since the use of substances of natural origin is perceived by patients as less invasive and safer. Herbal medicines still have (and still gain) many supporters in the modern world. Due to its pleasantly sweet flavor (a combination of apple and watermelon) and several possibilities of serving, Yacon roots are consumed willingly raw, boiled, roasted, or processed as vinegar, flour, beverages [7].

## 2. Materials and Methods

### 2.1. Plant Material

Mature tubers of *Smallanthus sonchifolius*–Yacon (*Asteraceae*) were the plant material used for the study. Yacon tubers were obtained from the Research Institute of Horticulture, Department of Applied Biology (Skierniewice, Poland) and confirmed by Agnieszka Dąbrowska from the Botanical Garden of Maria Curie-Sklodowska University (Lublin, Poland). A voucher specimen (No. 10_2019) was deposited in the Department of Pharmacognosy, Medical University of Lublin (Lublin, Poland). Cut tubers were dried at a temperature of 35 °C. After drying at room temperature, the plant material was cut into small pieces and pulverized in a laboratory grinder, and passed through a sieve with a mesh of 1.4 mm.

### 2.2. Accelerated Solvent Extraction

Accelerated solvent extraction (ASE), also called pressurized solvent extraction, was used to prepare Yacon tubers extract. Plant material samples (0.5 g each) were extracted in 10 mL extraction cell at default conditions (i.e., 80 °C, *p* = 90 bar, 3 static cycle, t = 10 min for each cycle) with methanol by the use of Dionex200 ASE apparatus (Dionex Corp., Sunnyvale, CA, USA). Obtained extracts were combined and transferred into 50 mL flat bottomed flasks and evaporated to dryness. The flask with the dry methanolic extract was placed for 12 h in a refrigerator at −70 °C and then lyophilized for 18 h.

ASE is a type of continuous extraction method, and the best device to describe its effectiveness is the yield, which amounted to 29.95%, resulting in 0.14975 g using a 0.5 g plant sample.

### 2.3. Drugs Administration

The whole Yacon tuber extract (25, 50, 100 mg/kg) and the tested antidepressants: imipramine hydrochloride (7.5 and 15 mg/kg, Sigma-Aldrich), fluoxetine hydrochloride (20 and 40 mg/kg, Sigma-Aldrich), and reboxetine mesylate (5 and 10 mg/kg, Sigma-Aldrich) were dissolved in saline. All the tested substances were administered intragastrically only once, 60 min before the behavioral tests. Animals in the control group received saline. A standard volume of liquid dosage forms (i.e., 10 mL/kg) was used. The pretreatment schedules and the tested doses were chosen on the basis of the literature data [29,34,35,36,37], and after that, they were confirmed under working conditions in our laboratory in preliminary studies and our previous experimental projects (unpublished data).

### 2.4. Animals

Male mice of the albino Swiss strain, weighing 28–30 g, were used to carry out the experiments. The cages with mice were located in air-conditioned rooms maintaining the ambient temperature within the range of 23–25 °C. The animals were provided with a natural 12 h day/night cycle, replicating the day and night mode. Each testing group was represented by 8–10 animals, depending on the research schedule. All experiments were approved by the Local Ethical Committee (no. of ethical approval: 21/2020).

### 2.5. Forced Swim Test (FST)

The forced swim test was developed by Porsolt and associates in 1977 [38]. It is a standard behavioral test, called the resignation test, which is used to determine the effectiveness of antidepressant drugs in laboratory rodents. According to this method, mice are put into glass cylinders (diameter 10 cm, height 25 cm) filled with water at room temperature (23–25 °C), and they are left there for 6 min. During the first 2 min of the test, the animals are supposed to adapt to the novel stressful environment. During the next 4 min, the time period in which animals remain motionless is measured using a stopwatch. After 6 min of the test, rodents are removed from the water, dried with a paper towel, and placed in their home cages, which should be located for a certain time near to a heating source.

### 2.6. Spontaneous Locomotor Activity

The measurement of the spontaneous locomotor activity was carried out using the OptoVarimex 4 Auto-Track device (Columbus Instruments, Columbus, OH, USA), according to the procedure we had used before [39]. Tested animals are kept in the cages for 6 min. Their activity was recorded as interruptions of the light beams between the 2nd and the 6th min of the test, and it was calculated automatically as a traveled distance in cm.

### 2.7. Statistical Analysis

Statistical analysis of the obtained results was performed by either one-way or two-way analysis of variance (ANOVA) with Dunnett’s or Bonferroni’s multiple comparisons test. The outcomes were given as the means ± standard error of the mean (SEM). Between-group differences with *p* lower than 0.05 were treated as statistically significant (where: * *p* < 0.05, ** *p* < 0.01, and *** *p* < 0.001).

## 3. Results

### 3.1. Effects of an Acute Administration of Yacon Tuber Extract in the FST in Mice

**Figure 1:**
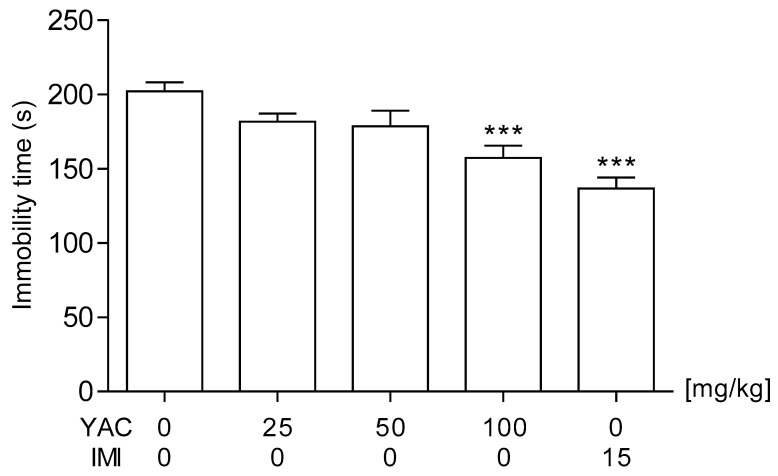
Effects of an acute administration of Yacon tuber extract in the forced swim test in mice. The Yacon extract (YAC; 25, 50, and 100 mg/kg), imipramine hydrochloride (IMI, 15 mg/kg), and saline were administered intragastrically 60 min before the test. The obtained data are presented as the means + SEM (*n* = 10 animals per group). *** *p* < 0.001 versus the saline-treated group (one-way ANOVA followed by Dunnett’s post-hoc test).

In order to determine the antidepressant potential of the Yacon extract, it was administered at the following intragastric doses: 25, 50, and 100 mg/kg. As was shown in Figure 1, the highest tested dose of the Yacon extract significantly reduced the total time of immobility of mice in comparison to the control saline-treated group (*p* < 0.001). Statistical analysis indicated that the lower doses (i.e., 25 and 50 mg/kg) did not exert any considerable effect in the FST (*p*  >  0.05). One-way ANOVA detected significant differences between the tested groups: *F*(4,49)  =  10.33; *p*  <  0.0001.

### 3.2. Effects of a Combined Administration of the Effective Doses of Yacon Tuber Extract and Antidepressants in the FST in Mice

**Figure 2:**
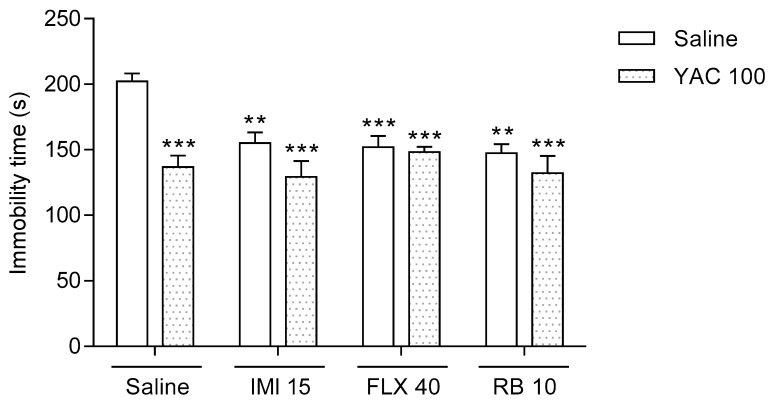
Effects of a combined administration of the effective doses of Yacon tuber extract and antidepressants. The Yacon extract (YAC, 100 mg/kg), imipramine hydrochloride (IMI, 15 mg/kg), fluoxetine hydrochloride (FLX, 40 mg/kg), reboxetine (RB, 10 mg/kg), and saline were administered intragastrically 60 min before the test. The obtained data are presented as the means + SEM (*n* = 8–10 animals per group). ** *p* < 0.01, *** *p* < 0.001 versus the saline-treated group (one-way ANOVA followed by Bonferroni’s post-hoc test).

As shown in Figure 2, the Yacon extract (100 mg/kg), imipramine hydrochloride (15 mg/kg), fluoxetine hydrochloride (40 mg/kg), and reboxetine mesylate (10 mg/kg) when given alone via oral gavage increased the time of mobility of animals in the FST when compared to the saline-treated control group (*p* < 0.01). Similarly, animals that received the Yacon extract (100 mg/kg) in combination with imipramine hydrochloride (15 mg/kg), fluoxetine hydrochloride (40 mg/kg), or reboxetine (10 mg/kg) presented an antidepressant-like behavior in the FST, i.e., their immobility time was significantly shorter when compared to the control group. Furthermore, Bonferroni’s post-hoc test did not indicate that the Yacon extract reduced the antidepressant activity of the tested drugs when given together. The immobility level of mice that received the tested Yacon-drug combinations was almost the same as the immobility level recorded for animals that were treated only with the Yacon extract or a respective antidepressant (*p* > 0.05).

### 3.3. Effects of a Combined Administration of the Sub-Effective Doses of Yacon Tuber Extract and Antidepressants in the FST in Mice

**Figure 3:**
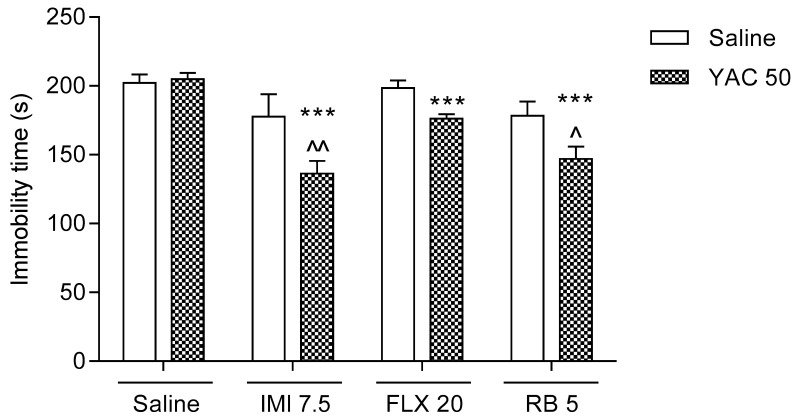
Effects of a combined administration of the sub-effective doses of Yacon tuber extract and antidepressants. The Yacon extract (YAC, 50 mg/kg), imipramine hydrochloride (IMI, 7.5 mg/kg), fluoxetine hydrochloride (FLX, 20 mg/kg), reboxetine mesylate (RB, 5 mg/kg), and saline were administered intragastrically 60 min before the test. The obtained data are presented as the means + SEM (*n* = 8–10 animals per group). *** *p* < 0.001 versus the Yacon-treated group; ^*p* < 0.05, ^^*p* < 0.01 versus the group treated with a respective antidepressant (one-way ANOVA followed by Bonferroni’s post-hoc test).

Neither the Yacon extract (50 mg/kg) nor imipramine hydrochloride (7.5 mg/kg), fluoxetine hydrochloride (20 mg/kg), or reboxetine mesylate (5 mg/kg), when given alone via oral gavage, influenced the behavior of animals subjected to the FST when compared to the saline-treated control group (*p* > 0.05). However, as was presented in Figure 3, intragastric administration of the Yacon extract in combination with imipramine hydrochloride (7.5 mg/kg), fluoxetine hydrochloride (20 mg/kg), or reboxetine mesylate (5 mg/kg) significantly reduced the immobility time in the FST in mice.

Two-way ANOVA indicated:

(A) A significant Yacon-imipramine interaction (F(1,34)  =  5.59, *p*  =  0.0240) with a significant effect of imipramine (F(1,34)  =  25.12, *p*  <  0.0001) and a significant effect of the Yacon extract (F(1,34)  =  4.25, *p*  =  0.0469);
(B) A significant Yacon-fluoxetine interaction (F(1,36)  =  6.26, *p*  =  0.0170) with a significant effect of fluoxetine (F(1,36)  =  10.49, *p*  =  0.0026) and a not significant effect of the Yacon extract (F(1,36)  =  3.78, *p*  =  0.0598);
(C) A significant Yacon-reboxetine interaction (F(1,35)  =  4.79, *p*  =  0.0355) with a significant effect of reboxetine (F(1,35)  =  27.37, *p*  <  0.0001) and a not significant effect of the Yacon extract (F(1,35)  =  3.35, *p*  =  0.0758).

### 3.4. Effects of an Acute Administration of Yacon Tuber Extract on the Spontaneous Locomotor Activity of Mice

As summarized in Table 1, none of the tested doses of the Yacon extract (i.e., 25, 50, 100 mg/kg) given intragastrically influenced the spontaneous locomotor activity of mice (*p* > 0.05).

### 3.5. Effects of a Combined Administration of the Effective Doses of Yacon Tuber Extract and Antidepressants on the Spontaneous Locomotor Activity of Mice

The Yacon extract (100 mg/kg), imipramine hydrochloride (15 mg/kg), fluoxetine hydrochloride (40 mg/kg), and reboxetine mesylate (10 mg/kg) administered via oral gavage either alone or in respective combinations did not exert statistically significant effects on the spontaneous locomotor activity in mice (*p* > 0.05) (Table 2).

Two-way ANOVA demonstrated:

(A) Not significant effect of imipramine (*F*(1,32)  =  0,02, *p*  =  0.8779), not significant effect of the Yacon extract (*F*(1,32)  =  0.27, *p*  =  0.6094), and no Yacon-imipramine interaction (*F*(1,32)  =  2.65, *p*  =  0.1136);
(B) Not significant effect of fluoxetine (*F*(1,32)  =  1.40, *p*  =  0.2458), not significant effect of the Yacon extract (*F*(1,32)  =  0.26, *p*  =  0.6158), and no Yacon-fluoxetine interaction (*F*(1,32)  =  0.76, *p*  =  0.3884);
(C) Not significant effect of reboxetine (*F*(1,34)  =  2.37, *p*  =  0.1329), not significant effect of the Yacon extract (*F*(1,34)  =  0.22, *p*  =  0.6448), and no Yacon-reboxetine interaction (*F*(1,34)  =  1.55, *p*  =  0.2222).

### 3.6. Effects of a Combined Administration of the Sub-Effective Doses of Yacon Tuber Extract and Antidepressants on the Spontaneous Locomotor Activity of Mice

As presented in Table 3, intragastrical administration of the Yacon extract (50 mg/kg), imipramine hydrochloride (7.5 mg/kg), fluoxetine hydrochloride (20 mg/kg), and reboxetine mesylate (5 mg/kg) either alone or in respective combinations did not significantly influence the spontaneous locomotor activity of mice.

## 4. Discussion

Products made from Yacon roots and leaves have found their place in the market of functional food in the form of tea, syrup, powder, and capsules. Yacon contains a range of biologically active components that produce beneficial health effects and may reduce the risk of developing several chronic diseases [40]. The possibility of using Yacon extracts in medicine has encouraged scientists to confirm their health properties in preclinical experiments and clinical trials.

Results of the present study for the first time demonstrated the antidepressant-like activity of the whole Yacon tuber extract. Albino Swiss mice that received an acute intragastric dose of 100 mg/kg were significantly much more active in the FST than their saline-treated counterparts. However, the lower tested doses of the Yacon extract (25 and 50 mg/kg) were not effective in the performed test. The obtained outcomes were in line with reports by An and associates [29], who found out that oral administration of the inulin-type oligosaccharides extracted from Yacon roots induced a considerable antidepressant-like effect in rodents. This activity was shown after an acute administration in mice (in the FST and in the tail suspension test) as well as after a subchronic (5-day) administration in rats (in the learned helplessness paradigm), and it was comparable to the one observed after treatment with antidepressant drugs (i.e., fluoxetine and duloxetine).

When examining whether ingestion of the whole Yacon tuber extract could influence the activity of antidepressant drugs, during the first stage of our experiments, we did not detect any antagonism in the antidepressant action of the used agents. Concurrent administration of the effective doses of the Yacon extract (100 mg/kg) with imipramine hydrochloride (15 mg/kg), fluoxetine hydrochloride (40 mg/kg), or reboxetine mesylate (10 mg/kg) did not reduce the level of mobility of the tested animals. Thus, we can presume that the main substances in the whole Yacon tuber extract should not have a negative impact on the absorption of antidepressant drugs. Results obtained during the second stage of our experiment demonstrated synergism in the antidepressant action of the whole Yacon tuber extract and the applied antidepressants. Mice given a combination of the sub-effective doses of the Yacon extract (50 mg/kg) and imipramine hydrochloride (7.5 mg/kg), fluoxetine hydrochloride (20 mg/kg), or reboxetine mesylate (5 mg/kg) were significantly more mobile in the FST than animals that received the same sub-effective dose of only one agent. Such an observation is very promising. The possibility of prescribing antidepressant drugs at lower doses than the ones used in monotherapy would minimize the risk of adverse reactions without negative effects on the treatment efficacy. It is particularly significant given the safety profile of Yacon preparations. Reports from clinical trials confirmed that Yacon products are generally well tolerated by patients [23,25,26,41], though the daily intake of Yacon syrup containing 20 g of fructooligasaccharides/70 kg body weight/day may induce gastrointestinal adverse effects, including diarrhea, severe abdominal distention, flatulence, and nausea [23]. One case report on the anaphylactic reaction due to ingestion of Yacon root can be found in the medical literature [42].

In order to respect the 3R rules (replacement, reduction, and refinement) for assessment of the antidepressant potential of the Yacon extract and its co-administration with antidepressant drugs, we applied only one behavioral test in the present study, i.e., the FST. Though the mouse FST test is believed not to be consistently sensitive for detecting SSRI activity [43,44], in our previous studies, we demonstrated comparable results in the FST and in the TST related to fluoxetine and escitalopram treatment. Sometimes the observed effect was more pronounced in the TST, but it was not a rule (e.g., [45,46,47,48,49]). Thus, it can be concluded that under our laboratory conditions, similar effects are obtained in the FST and in the TST in albino Swiss mice for SSRIs. Therefore, in our opinion, the application of the FST only (accompanied by the measurement of the spontaneous locomotor activity) should be enough to suggest that the Yacon extract could be promising as an alternative mood-improving product. The obtained actinometer readings allow us to be sure that the observed behavior of mice in the FST was not due to the treatment-induced increase in their mobility but was caused by the antidepressant potential of the Yacon extract and the applied drug combinations. In the present study, we did not detect any significant differences in the spontaneous locomotor activity between the tested groups. Our results were in line with outcomes reported by An et al. [29], who also did not show any stimulatory effects of the inulin-type oligosaccharides extracted from Yacon roots on the locomotor activity of mice.

Though our experiments did not assess the molecular mechanism of the antidepressant-like activity of Yacon tuber extract, we guess that it can be at least partially caused by the antioxidant [10,41], anti-inflammatory, and probiotic properties of its main components, particularly fructooligosaccharides, inulin, polyphenols, and tryptophan. It would be in line with new theories of depression, which associate the development of this disease with oxidative stress [50], (neuro)inflammation [51], and disturbances in the gut-microbiota-brain axis [52]. In diabetic rats, Yacon supplementation resulted in a considerable reduction in the hepatic and renal levels of malondialdehyde and activities of superoxide dismutase and catalase. Additionally, it elevated values of glutathione peroxidase and glutathione in the liver and kidneys, and it normalized the composition of liver fatty acids along with cholesterol and triacylglycerol levels in plasma [53]. In the double-blind placebo-controlled clinical trial, a 6-week consumption of Yacon flour resulted in antioxidant activity, and it increased the plasma total antioxidant capacity [41]. In the recent study by Baek et al. [54], it was demonstrated that the Yacon leaf extract produced a significant neuroprotective effect in the lipopolysaccharide-induced mouse model of neuroinflammation by reducing mRNA expression of the inflammatory factors, including inducible nitric oxide synthase, cyclooxygenase-2, interleukin 1β, and tumor necrosis factor (TNF)-α. A 16-week supplementation with Yacon flour also improved the plasma TNF-/interleukin-10 ratio (representing a balance between pro- and anti-inflammatory cytokines) in a rat model of colorectal carcinogenesis [55]. According to results obtained in preclinical studies, fructooligosaccharides from Yacon can increase production of the short-chain fatty acids, and in consequence, they can strengthen the gut immune responses and suppress the local inflammation [9,56]. Similarly, inulin, that is also a component of Yacon root, seems to promote short-chain fatty acid production [57]. It has been demonstrated that a prolonged administration of the Yacon root flour stimulated the growth of the intestinal *Bifidobacteria* spp. and *Lactobacillus* spp. in murine [58,59], Gallus gallus [60], and guinea pig [61] models, as well as it improved the intestinal immune responses [58,59], whereas lower amounts of *Bifidobacterium* and/or *Lactobacillus* were detected in the microbiota of patients with major depression [62]. Quite recently, several authors have reported beneficial effects of *Lactobacillus* spp. and/or *Bifidobacterium* supplementation on depressive symptoms in adults [63,64,65,66].

## 5. Conclusions

In conclusion, results obtained in the present study are promising as they indicate the antidepressant potential of the whole Yacon tuber extract and its ability to act synergistically with typical antidepressant drugs. Our observation certainly requires confirmation in further, more extensive experiments, but it suggests that the whole Yacon tuber extract could find its place among unconventional mood-improving products in the future.

## Figures and Tables

**Table 1:** Effects of an acute administration of Yacon tuber extract on the spontaneous locomotor activity of mice.

Treatment (mg/kg)	Traveled Distance (cm)	Number of Animals Per Group
Saline	484.0 ± 56.78	10
Yacon 25	462.3 ± 57.72	10
Yacon 50	429.3 ± 53.96	10
Yacon 100	550.0 ± 51.22	10
Imipramine 15	525.0 ± 65.21	10

The Yacon extract (25, 50, and 100 mg/kg), imipramine hydrochloride (15 mg/kg), and saline were administered intragastrically 60 min before the test. The obtained data are presented as the means ± SEM (one-way ANOVA followed by Dunnett’s post-hoc test).

**Table 2:** Effects of a combined administration of the effective doses of Yacon tuber extract and antidepressants on spontaneous locomotor activity of mice.

Treatment (mg/kg)	Traveled Distance (cm)	Number of Animals Per Group
Saline + saline	519.80 ± 46.3	10
Yacon 100 + saline	590.70 ± 23.7	10
Imipramine 15 + saline	568.50 ± 24.2	8
Yacon 100 + imipramine 15	531.75 ± 24.9	8
Fluoxetine 40 + saline	504.00 ± 61.6	8
Yacon 100 + fluoxetine 40	485.12 ± 72.3	8
Reboxetine 10 + saline	507.50 ± 46.5	8
Yacon 100 + reboxetine 10	475.20 ± 45.5	10

The Yacon extract (100 mg/kg), imipramine hydrochloride (15 mg/kg), fluoxetine hydrochloride (40 mg/kg), reboxetine (10 mg/kg), and saline were administered intragastrically 60 min before the test. The obtained data are presented as the means ± SEM (*n* = 8–10 animals per group) (two-way ANOVA followed by Bonferroni’s post-hoc test).

**Table 3:** Effects of a combined administration of the sub-effective doses of Yacon tuber extract and antidepressants on spontaneous locomotor activity of mice.

Treatment (mg/kg)	Traveled Distance (cm)	Number of Animals Per Group
Saline + saline	517.30 ± 46.9	10
Yacon 50 + saline	522.40 ± 64.1	10
Imipramine 7.5 + saline	523.87 ± 39.0	8
Yacon 50 + imipramine 7.5	562.00 ± 22.3	9
Fluoxetine 20 + saline	451.40 ± 45.1	10
Yacon 50 + fluoxetine 20	495.30 ± 22.5	10
Reboxetine 5 + saline	362.80 ± 32.5	10
Yacon 50 + reboxetine 5	381.30 ± 31.3	10

The Yacon extract (50 mg/kg), imipramine hydrochloride (7.5 mg/kg), fluoxetine hydrochloride (20 mg/kg), reboxetine (5 mg/kg), and saline were administered intragastrically 60 min before the test. The obtained data are presented as the means ± SEM (two-way ANOVA followed by Bonferroni’s post-hoc test).

## Data Availability

The data presented in this study are available on request from the corresponding author.
